# Comparative role of neem seed extract, moringa leaf extract and imidacloprid in the management of wheat aphids in relation to yield losses in Pakistan

**DOI:** 10.1371/journal.pone.0184639

**Published:** 2017-09-27

**Authors:** Farhan Mahmood Shah, Muhammad Razaq, Abid Ali, Peng Han, Julian Chen

**Affiliations:** 1 Department of Entomology, Faculty of Agricultural Sciences and Technology, Bahauddin Zakariya University, Multan, Pakistan; 2 Department of Entomology, University of Agriculture, Faisalabad, Pakistan; 3 INRA (French National Institute for Agricultural Research), Univ. Nice Sophia Antipolis, CNRS, UMR 1355-7254, Institut Sophia Agrobiotech, Sophia Antipolis, France; 4 The State Key Laboratory for Biology of Plant Diseases and Insect Pests, Institute of Plant Protection, Chinese Academy of Agricultural Sciences, Beijing, China; Institut Sophia Agrobiotech, FRANCE

## Abstract

Wheat being staple food of Pakistan is constantly attacked by major wheat aphid species, *Schizaphis graminum* (R.), *Rhopalosiphum padi* (L.) and *Sitobion avenae* (F.). Due to concern on synthetic chemical use in wheat, it is imperative to search for alternative environment- and human- friendly control measures such as botanical pesticides. In the present study, we evaluated the comparative role of neem seed extract (NSE), moringa leaf extract (MLE) and imidacloprid (I) in the management of the aphid as well as the yield losses parameters in late planted wheat fields. Imidacloprid reduced significantly aphids infestation compared to the other treatments, hence resulting in higher yield, particularly when applied with MLE. The percentages of yield increase in I+MLE treated plots over the control were 19.15–81.89% for grains per spike, 5.33–37.62% for thousand grain weight and 27.59–61.12% for yield kg/ha. NSE was the second most effective control measure in suppressing aphid population, but the yield protected by NSE treatment over the control was comparable to that by imidacloprid. Population densities of coccinellids and syrphids in the plots treated with NSE-2 were higher than those treated with imidacloprid in two out of three experiments during 2013–14. Low predator density in imidacloprid-treated plots was attributed to the lower availability of prey aphids. The efficacy of NSE against aphids varied depending on degree of synchronization among the application timing, the activity of aphids, crop variety and environmental conditions. Despite that, we suggested NSE to be a promising alternative botanical insecticide compared to the most commonly recommended imidiacloprid. Further studies should consider the side effects of biopesticides on non-target organisms in order to provide better management practices in the field.

## Introduction

Over the decades, pesticides have been widely applied as a standard practice to control agricultural pests in the field [1, 2]. Their constant use has caused selection of resistances in agricultural pests, environmental pollution with negative side effects on human health and on non-target arthropods [1–4]. Among the diverse environmental-friendly and safe strategies for pest management, biopesticides represent one of the best alternatives to chemicals [5]. These compounds have various physiological and behavioral effects on insect pests [6]. The insecticidal activity of botanicals is well known for their use for thousands of years worldwide in all the agricultural regions [7]. Botanical insecticides are generally less harmful to the environment and their use avoids the development of insect resistance [8–10]. Active substances in botanical insecticides degrade easily and rapidly through natural degradation processes [11]; further the presence of multiple active ingredients that act synergistically and exhibit various mode of action prevents resistance developments in pest populations [12]. Botanical insecticides, being cheap, effective, safe, easy to process and to apply, can also represent the best option in developing countries for farmers in the insect pest management [13, 14]. However, integrated use of biopesticides with the bio-control agents may not cause acute effects but could induce sublethal effects (e.g., physiological and behavioral) which consequences a decrease in population growth of biocontrol services [15–17].

Wheat *Triticum aestivum* L. being a staple food, shares 9.9 and 2% to the value addition in agriculture and GDP in Pakistan, respectively [18]. Wheat crop is planted beyond November 20^th^ due to late harvest of cotton in cotton-wheat cropping system in Asia [19]. Many researchers have documented yield losses in response to temperature stress and late wheat planting [20–22]. To delay early maturity or leaf senescence in late sown wheat, an effective approach is to use plant growth regulators (PGRs) that contain cytokinins [23, 24]. Cytokinins enable plants to grow well under normal as well as stress circumstances [25]. Commercially available PGRs are quite expensive. Late sown wheat becomes more susceptible to aphid attack [26, 27] as well as to other important insect pests [28, 29]. Response of aphids varies with respect to sowing time [30, 31]. Aphid inflicts significant economic losses to wheat and other cereals by direct feeding on phloem sap [32, 33] or indirectly by carrying and spreading plant viruses, especially barley yellow dwarf virus (BYDV) between crops [34]. Moreover, secretion of honeydew on leaves interferes with photosynthetic and respirational functions of plants and consequently boosts leaf senescence [35]. Among the aphids, *Sitobion avenae* (F.), *Rhopalosiphum padi* (L.) and *Schizaphis graminum* (R.) (Hemiptera: Aphididae) are major insect pests of wheat in Pakistan [36, 37] and other parts of sub-continents [38, 39]. Thus, the effective control of these pests is desired to minimize yield losses and to enhance net returns.

Aphids can be managed successfully with either neonicotinoid insecticide like imidacloprid applied as seed treatment [40] or foliar applications [41, 42]. Imidacloprid, a neonicotinoid insecticide, owing to systemic nature is translocated throughout the plant parts following application and effective as poison on contact as well after ingestion [43]. Neonicotinoid binds in the nicotinic acetylcholine receptors site in the post-synapse, following spontaneous discharge leading to failure of nerve impulse propagation throughout of neuron [44]. Neonicotinoids are the widely used class of insecticides worldwide, however, their persistence and accumulation in soil, water ways, pollen and nectar of treated crops is the leading environmental concern to a range of ecosystem services including beneficial organisms, pollinators, soil and aquatic invertebrates [45]. Therefore, it is imperative to search for environment- and human- friendly alternatives. Seeds of neem tree, *Azadirachta indica*, A. Juss. (L.) (family Meliaceae) are rich in extractable compounds like azadirachtin [46], a tetranotriterpenoidlimonoids known to have antifeedant and growth disruptive effect on more than 540 insect species [47]. Many studies reported its effective use in managing different insect pests [48–50]. Besides azadirachtin, the seeds contain more than a dozen of their analogs or other triterpenoids notably nimbin, salannin and their derivatives. However, these triterpenoids contribute little towards efficacy of extract [51]. Azadirachtins are applied as aqueous, alcoholic and azadirachtin enriched extracts. Their residual activities last for 4–8 days, depending upon treated plant species and surrounding environmental conditions; the efficacy lasts slightly longer if applied systemically [52]. Botanical insecticide has long been considered as an attractive alternative to synthetic insecticide for management of pests because the former poses little threat to environment compared to the latter. Plants based essential oils like pyrethrum and neem have stepped in market place and are best suited for organic food production in developing and industrial countries [53]. Several plants extract with different mode of action have potential to be employed in pest management. Azadirachtin, extracted from neem seeds, acts on target organism as antifeedent, growth inhibitor and insect growth regulator [54]. Akhtar et al. [54] reported that plant species of the family Meliaceae such as *Azadirachta indica*, *A*. *excelsa*, *Trichilia americana* and *Melia volkensii* are rich sources of active botanical insecticides. Their level of activity, based upon growth inhibition, chronic toxicity, and antifeedant activity, can be compared favorably to some of the commercialized botanical products. Moreover, essential oils including those obtained from citrus fruit peel of Rutaceae family have potential to be employed in pest management programs against stored grain pests, fumigant nature of oil, at low concentration, even at the end of fumigation caused increased mortality to *Tribolium confusum* Du Val, (Coleoptera: Tenebrionidae) [55]; combining sweet orange [*Citrus sinensis* (L.) Osbeck] peel essential oil with kaolin had synergistic effect on mortality of *Rhyzopertha dominica* (F.) (Coleoptera: Bostrichidae) [56]. Sandalwood oil, (*Santalum austrocaledonicum* Vieill), (family Santalaceae) main components i.e. a- and b-santalol had repellent as well as insecticidal effect against *Aphis gossypii* Glover (Hemiptera: Aphididae) [57]. However, the scope of plant essential oils and/or extracts is not only limited to use against agricultural insect pests [58] but also against hematophagic insect pests of medical and veterinary [59], and urban environments [60].

In addition, the extracts obtained from moringa leaves, *Moringa oleifera* Lam. (family Moringaceae) play important role in enhancing crop growth and yield under stress [61, 62]. Moringa tree is widely cultivated in tropical and subtropical parts of the world. Aqueous extracts obtained from moringa seeds (which contained water soluble *M*. *oleifera* seed lectin (WSMoL)) and moringa flowers (which contained secondary metabolites: β-amyrin, β-sitosterol, kaempferol, and quercetin) against larvae of *Aedes aegypti* (L.) (Diptera: Culicidae) showed bioinsecticidal potential by creating morphological alternation in digestive tract, misbalancing digestive enzymes [63] and trypsin inhibitor activities [64], and interfering with egg hatchability [65, 66]. Moreover, seeds (which contained coagulant *M*. *oleifera* lectin (cMoL)) and pulverized leaves of *M*. *oleifera* showed bioinsecticidal potential by delaying digestion, and by disturbing the molting in *Anagasta kuehniella* (Zeller) (Lepidoptera: Pyralidae) and *Tribolium castaneum* (Herbst.) (Coleoptera: Tenebrionidae), respectively [67, 65]. The objectives of present study were to assess the effect of sowing time on aphid abundance and to evaluate the comparative effects of neem seed extract (NSE), moringa leaf extract (MLE) and imidacloprid on the management of wheat aphids, their arthropod natural enemies and wheat yield parameters.

## Materials and methods

### Field experimental setup

Experiments were conducted in two growing seasons from 2012–2013 (equal to 2012 in figures) to 2013–2014 (equal to 2013 in figures) at the research farm of Bahauddin Zakariya University (hereafter read as BZU), Multan, Punjab Province of Pakistan (30° 11' 44" N / 71° 28' 31" E.). Wheat plots were located in fields that had long history of wheat cultivation in rotation with cotton. Annually, three experiments were conducted by sowing wheat varieties “Punjab 2008” on November 27^th^, December 6^th^ and December 16^th^ in 2012–13 and “AARI 2011” on November 24^th^, November 27^th^ and December 3^rd^ in 2013–14 wheat growing season. Soil texture was clay loam. Seeds of both varieties at rate of 110kg/ha were sown in 25cm spaced wheat rows by single row hand drill. Nitrogen and Phosphorus fertilizers were applied at rate of 120 and 100 kg per hectares, respectively, with urea and single super phosphate (SSP) being the source of fertilizers. From fertilizers, whole phosphorus (P) while 1/3^rd^ of nitrogen (N) was applied as basal dose. However, rest of N was applied in two splits with 1^st^ and 2^nd^ irrigation. Each experimental plot area was 12m×25m and divided into three blocks (replications). Individual blocks were split into experimental units. All experiments were laid out in randomized completely block design with seven treatments (three replicates each). The seven treatments were as followed: (1) one spray of neem seed extract, NSE-1; (2) two sprays of neem seed extract, NSE-2; (3) imidacloprid, I; (4) moringa leaf extract, MLE; (5) imidacloprid + moringa leaf extract, I+MLE; (6) MLE + NSE; and (7) control, no spray. Area of every experimental unit was 5m×3m with 1m distance between treatments and replications to avoid spray drift.

### Preparation of plant extracts

MLE was prepared from fresh leaves of mature moringa tree located at biopark of BZU, Multan, Pakistan. Selected young sprouts on moringa tree were tagged and 38–40 days old leaves were plucked, packed in resealable plastic bags and shifted to laboratory. Leaves were washed with tap water to remove dust and dried at room temperature. Extract was prepared by following the method of Basra et al. [68]. Briefly, fresh moringa leaves were ground in a national electric blender (Moulinex^®^, model 276) by adding water in the ratio of (1L water/10 kg of fresh materials). The blend was sieved through muslin cloth to obtain water extract that was further centrifuged to get extract free from moringa leaf suspensions. To prepare NSE, fresh neem seeds were obtained from neem trees located at university colony—C4, BZU, Multan, Pakistan during July 2011. Neem seeds after maturation usually fell down on ground. Collected seeds were shade dried at room temperature, depulped, and washed with tap water to remove surface residues. NSE was prepared by following the method of Malian farmers as depicted by Boursier et al. [69]. Briefly, neem seeds were ground using national electric blender (Moulinex^®^, model 276). A 100 g of ground neem seeds soaked in 1L water for 3–7 days yielded NSE (10% w/v).

### Field application of botanicals and imidacloprid

Imidacloprid (Confidor, 20% SL, Bayer Crop Science) was obtained from insecticide resistance laboratory of Department of Entomology, Faculty of Agricultural Sciences and Technology (hereafter read as FAST), BZU, Multan, Pakistan. Foliar treatment with imidacloprid was applied at rate of (a.i 98.8 ml/ha) with a hand operated 19.5 L knapsack sprayer (PB-20; Cross Mark Sprayers, Johor, West Malaysia) and fitted with hollow cone nozzle. NSE (10% w/v) was further diluted at 5% level by mixing water in it prior to application in field by dividing the quantity/amount of water required to spray NSE plots (three plots/treatment) by hundred and multiply it by five. The obtained value was the amount of NSE (10% w/v) and added into required amount of water to yield NSE at 5% level (50ml NSE/1L of water). MLE extract prior to field application was further diluted in water at ratio (1:30) by mixing one part of MLE in thirty parts of water (33.3 ml/IL of water). Separate sprayer tanks were used for all treatments. Imidacloprid was applied at rate of 0.33 ml a.i/1L of water. Through calibration in a non-experimental plot, seven liter water was determined to spray an individual treatment with its replicated plots.

All treatments were applied at heading stage (characterized by appearance of erected heads on tillers and was not covered by leaf sheath) for each season. Heading stage commenced between 11-13^th^ March and 8-10^th^ March in 2012 and 2013 seasons, respectively. MLE and imidacloprid was applied on March 13^th^ and 14^th^, respectively, imidacloprid alone on March 13^th^, MLE alone on March 14^th^, NSE-1 on 13^th^ March, while NSE-2 on 13^th^ and 18^th^ March and NSE and MLE on March 13^th^ during the season 2012. In the season 2013, MLE and imidacloprid was applied on March 10^th^ and 11^th^ respectively, MLE alone on March 10^th^, imidacloprid alone on March 11^th^, NSE and MLE on March 10^th^, NSE-1 on 10^th^ March and NSE-2 on 10^th^ and 18^th^ March. Combined treatments were applied solely rather than tank mixture.

### Sampling

All wheat aphids regardless to species during 2012 were counted from five randomly selected plants per locations (overall three locations) per plot at seven-day intervals starting from March 9^th^ to 30^th^. Same sampling plan was practiced for the experiment in 2013, except that the samples were recorded at four-day intervals. The guild of natural enemies such as coccinellids, syrphids, spiders and parasitoid aphids were also counted. Counts of aphid species and natural enemies from the same plant (three locations per plot, five plants per location) were recorded separately on March 10^th^, 14^th^, 18^th^, 22^nd^, 26^th^ and 30^th^ (Table 1). *Schizaphis graminum*, *R*. *padi* and *S*. *avenae* were the most common wheat aphid species. The aphid species were morphologically identified using the identification keys of Blackman and Eastop [70]. Voucher specimens and data sheets were stored in the IPM laboratory at the Department of Entomology, FAST, BZU, Multan, Pakistan. The population density of parasitoids was estimated by counting the Aphidiinae mummies per plant. The mummies were separated by color basis like tan colored (Aphidiinae) and black (Aphelinidae) [71]. The specimens of wheat aphid species and natural enemies were collected and brought to laboratory. The samples were kept in separate plastic jars 25±1°C temperature and 65±5% R.H. Predators were identified to family level from Zoological Department of BZU, Multan, Pakistan.

**Table 1:** Total counts of wheat aphids and natural enemies observed in the control plots of wheat fields planted on 24^th^ November,27^th^ November and 3^rd^ December during the growing season of 2013.

Insect guild	Species	Sowing dates		
		24^th^ Nov.	27^th^ Nov.	3^rd^ Dec.
Aphids	*S*. *graminum*	16968	26280	12898
	*R*. *padi*	1064	4500	272
	*S*. *avenae*	2924	1088	2140
Natural enemies	Coccinellids	284	186	132
	Syprphids	24	10	40
	Spiders	14	6	8
	Aphid parasitoids	208	64	208

### Yield losses

During 2012–13, wheat at maturity was harvested on April 22^nd^ (planted on November 27^th^), April 27^th^ and 29^th^ (planted on December 6^th^ and 16^th^, respectively). The harvesting was carried out on April 24^th^ on the wheat that planted on all the three dates in 2013–14. In the middle of every plot to avoid the possible effect of nearby treatment on yield, a quadrate measuring 1m×1m (used as a standard to estimate yield per hectares) was fitted, and tillers covered inside were harvested and tagged with the name of respective plot (treatment/replicate). Yield/m^2^ was obtained after threshing manually beating with wooden stick until all the grains from spikes were collected inside the sacks and packed in plastic bags. Yield obtained was per meter sq. and converted to per hectares by multiplying number of meter sq. per hectares, i.e. 10000 m^2^. To measure the effect of treatments on yield over control, the yield difference between each treated and untreated plot was calculated by subtracting the yield of treated plot from untreated plot. The difference obtained was divided by yield of untreated plot, and multiplied by 100 to express yield difference in percentage. The same formula was used for calculating the percentage yield difference between treated and untreated plots in terms of shoot biomass, grains/spike and thousand grain weight. In addition, 30–35 tillers per plot were harvested and tagged with the information of respective plot and transferred to laboratory. Shoot biomass (in gram) was recorded by weighing individual tiller on electric balance with ten tillers per plot per replicate. The numbers of grains per spike were calculated by manual removing of grains from ten spikes per plot per replicate. Thousand-grain weight (in gram) was estimated by weighing one thousand wheat grains from the tillers.

### Statistical analysis

Prior to analysis, data of aphids, predators and predator-prey ratio were tested for normality and homoscedasticity of variance using Shapiro-Wilk and Levene tests (SPSS version 21). Only non-normal data were transformed to homogenize variance using equation Y = √ x+1 [72]. However, original means were presented in tables and figures rather than transformed values. To observe the effect of sowing dates on aphid abundance, data from untreated plots were analyzed with repeated measures ANOVA where sowing times represented a between-subject factor and sampling dates a within-subject factor. On significance (*P*<0.05), means were compared using LSD test. Seasonal counts of all predators per plant were summed across all the sampling dates. Similarly, seasonal counts of predator-prey ratios were also summed up across the sampling dates. Significant effect of treatments (*P*<0.05) on total sum or means (±SEM) of aphids, predators, predator-prey ratio, yield and its parameters were calculated via ANOVA using GLM, in SPSS (version 21). Means after significance were separated by Tukey’s HSD test. All graphs were made by Graphpad Prism 6.

## Results

### Insect guild in the control plot during 2013

During the 2013 season, insect guild in the control plot consisted of wheat aphids (*S*. *graminum*, *R*. *padi* and *S*. *avenae*) and natural enemy assemblages including coccinellids, mainly *Coccinella septempunctata* Linnaeus and *Coccinella undecimpunctata* Linnaeus (Coleoptera: Coccinellidae), syrphids, mainly *Ischiodon scutellaris* (Fabricius) (Diptera: Syrphidae), spiders, mainly *Oxyopes javanus* Thorell (Araneae: Oxyopidae) and *Pardosa birmanica* Simon (Araneae: lycosidae) and aphid parasitoids, mainly emerged primary parasitoids were *Aphidius colemani* Viereck (Hymenoptera: Braconidae) (Table 1). Among aphids, *S*. *graminum* (26280) was more numerous and considerably on November 27^th^ planted wheat followed by November 24^th^ (16968) and December 3^rd^ (12898) wheat crops. *Sitobion avenae* dominated over *R*. *padi* in November 24^th^ and December 3^rd^ planted wheat while vice versa on November 27^th^ planted wheat crops. Among natural enemy assemblages, the most dominated guilds were coccinellids, followed by aphid parasitoids (Table 1).

### Comparative effect of sowing dates on aphid abundance

**Fig 1:**
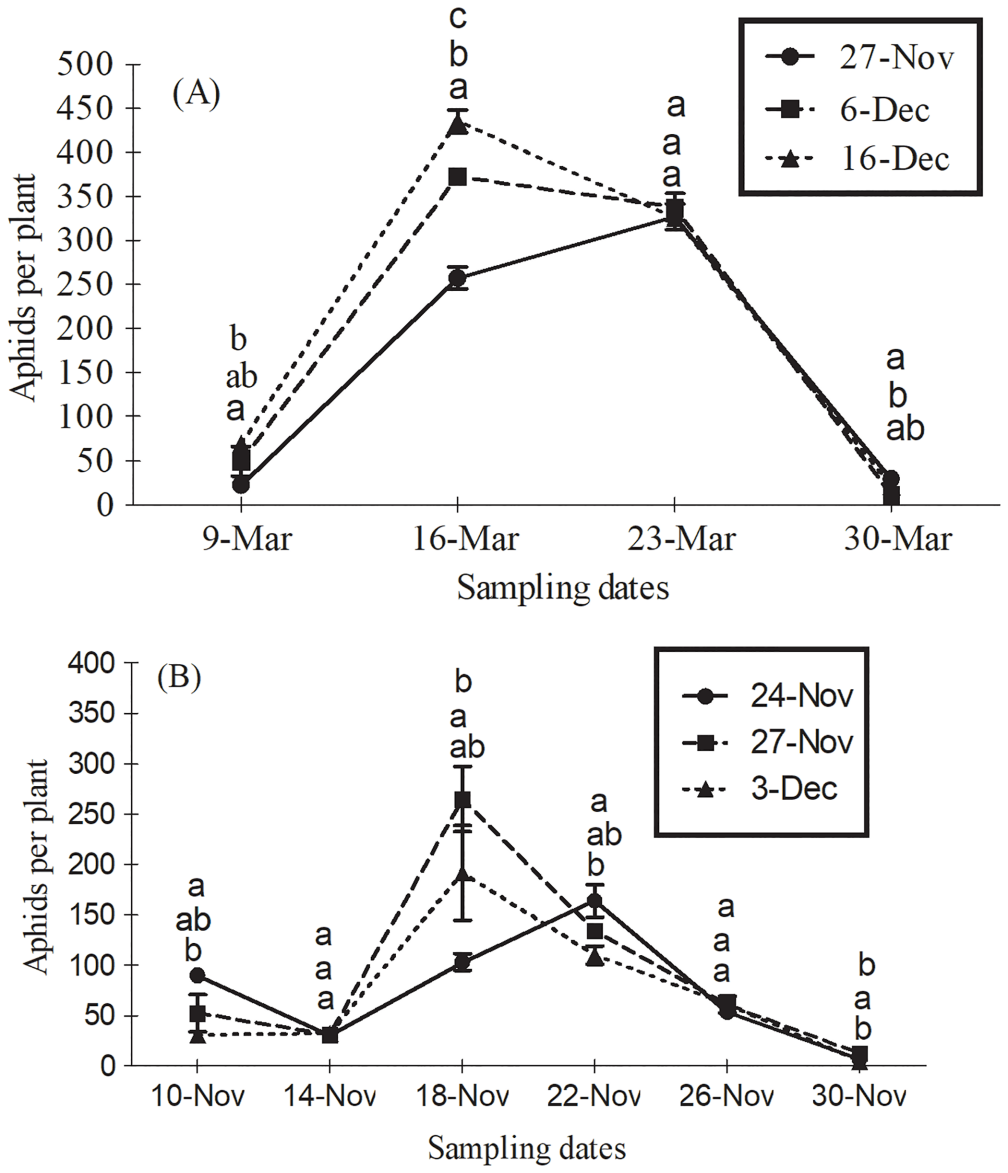
Mean (± SE) numbers of wheat aphids per plant on several observation dates between crops planted at different sowing times during the growing season of (A) 2012 and (B) 2013. Wheat was planted on 27^th^ November, 6^th^ and 16^th^ December in 2012; and on 24^th^ and 27^th^ November and December 3^rd^ in 2013. Means within each observation date sharing common letters are not statistically different at *P* < 0.05 (LSD test).

In 2012 season, response of aphids varied significantly in relation to planting time (*F*_2,6_ = 56.67; *P*<0.001) on several observation dates (*F*_6,18_ = 15.82; *P*<0.001). Aphids were more in December 16^th^ planted wheat followed by December 6^th^ and November 27^th^ planted wheat (Table 2). Aphid population reached to peak a week later on March 23^rd^ in November 27^th^ wheat than did in other planting times (Fig 1A). In 2013 season, planting time had no impact on aphid abundance among wheat crops (*F*_2,6_ = 2.33; *P* = 0.17). However, their population varied significantly among planting dates on several observation dates (*F*_10,30_ = 6.81; *P*<0.001). Generally, aphids were lowest in November 24^th^ planted wheat (Table 2). Peak activity was observed on March 18^th^ in November 27^th^ and December 3^rd^ planted wheat while on March 22^nd^ in November 24^th^ planted wheat (Fig 1B).

**Table 2:** Effect of sowing dates on mean (± SE) numbers of wheat aphids during the growing season of 2012 and 2013.

Year	Sowing date^b^	Aphids^a^
2012	27-Nov	159.02±3.00 c
	6-Dec	192.51±2.56 b
	16-Dec	212.8±4.83 a
2013	24-Nov	74.78±1.78 a
	27-Nov	92.87±8.13 a
	3-Dec	72.05±9.74 a

^a^ Mean number of aphids per plant.

^b^ Wheat was planted at three sowing dates in each year.

Means in column followed by different letters within a year are statistically different at *P* < 0.05 (LSD test).

### Impact of imidacloprid and botanicals on wheat aphids

**Fig 2:**
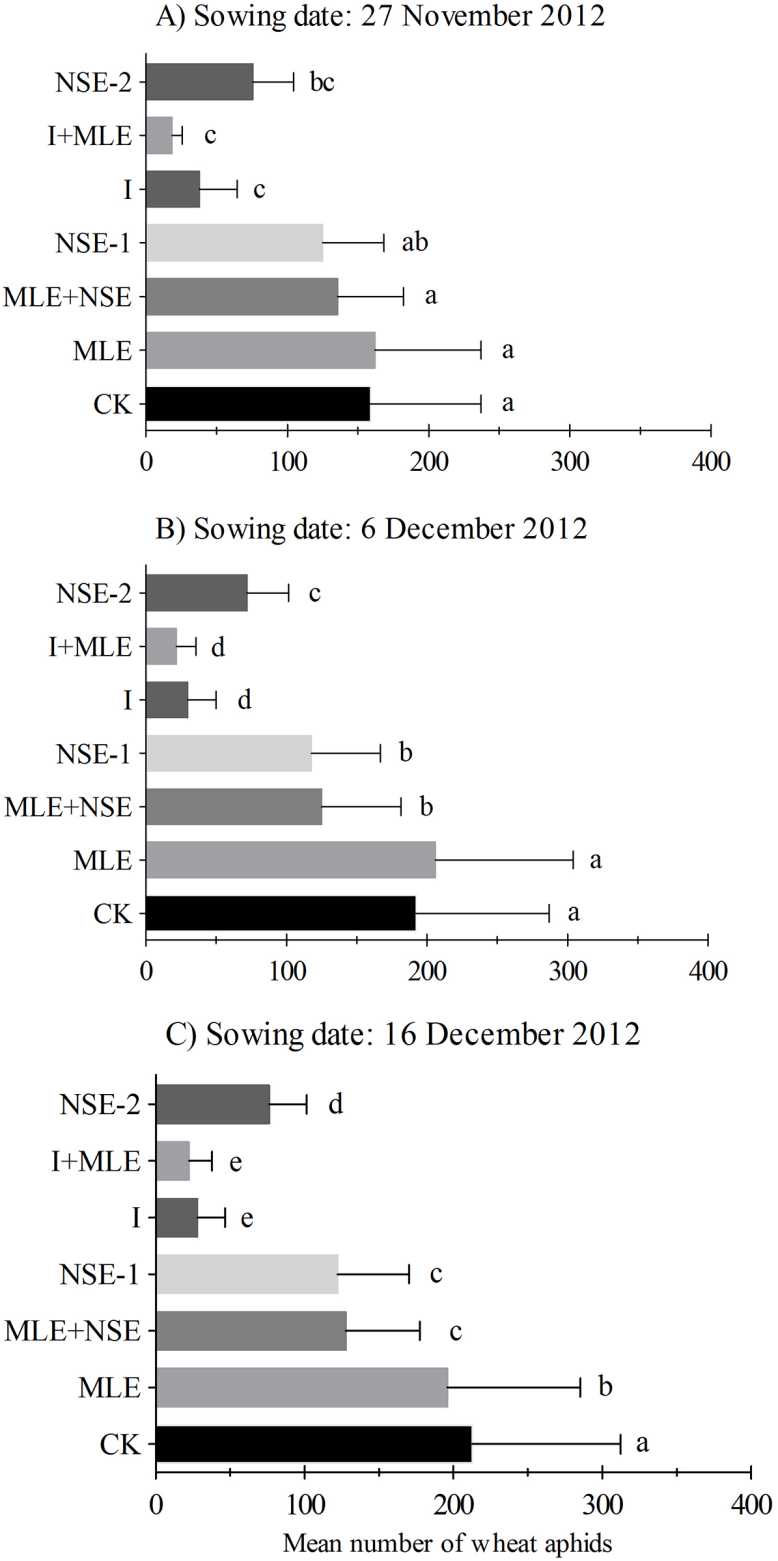
Mean (± SEM) number of wheat aphids per plant in the growing season 2012–2013. Wheat was planted on dates (A) 27^th^ November (B) 6^th^ December, (C) 16^th^ December. Control: no spray; MLE: spray of moringa leaf extract; MLE+NSE: spray of moringa leaf extract in combinaiton with neem seed extract; NSE-1: one spray of neem seed extract; I: spray of imidacloprid; I+MLE: spray of imidcloprid in combination with moringa leaf extract; NSE-2: two sprays of neem seed extract. Means on a given sowing date sharing common letters are not statistically different at *P* < 0.05 (Tukey’s HSD test).

**Fig 3:**
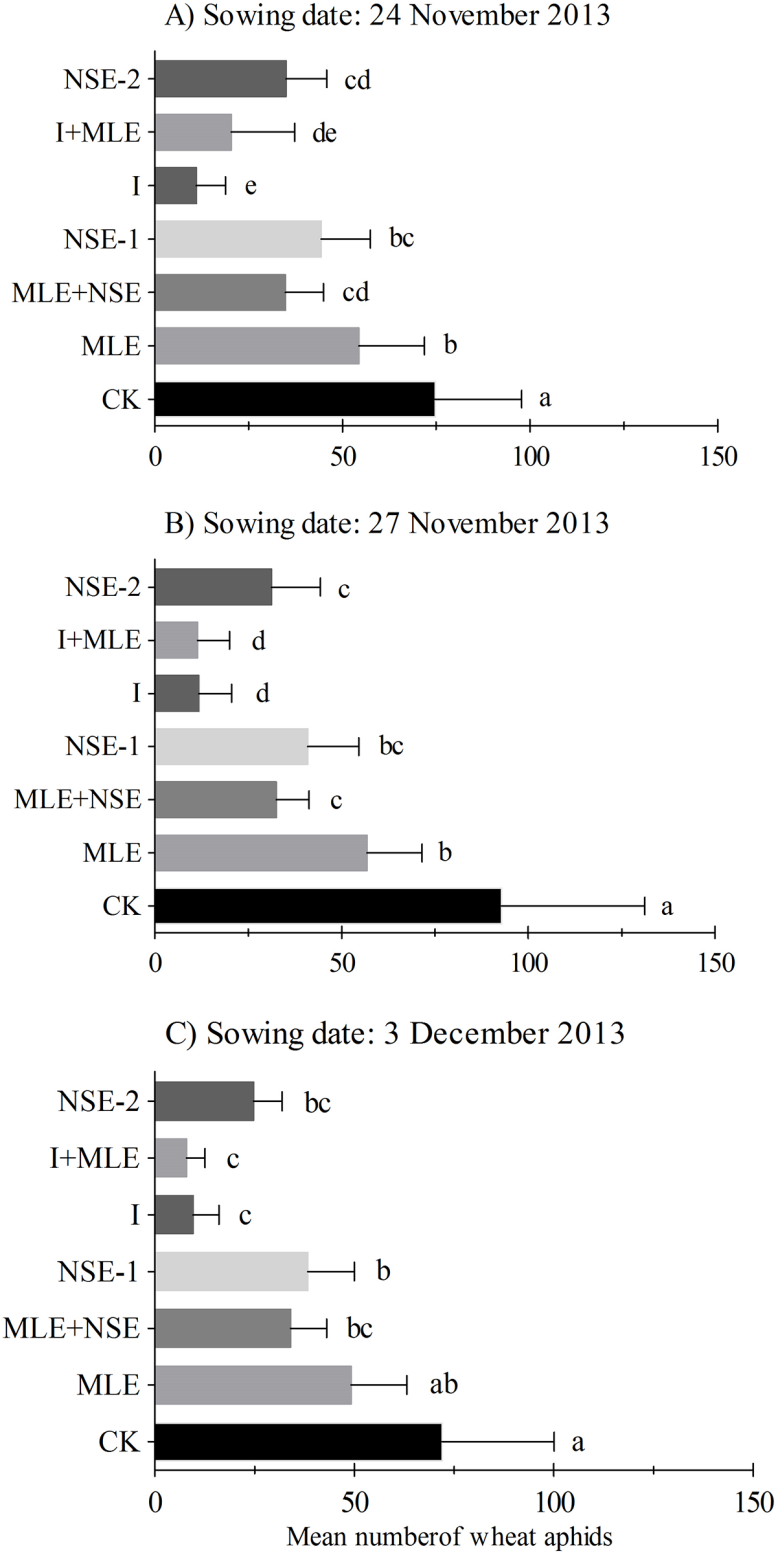
Mean (± SEM) number of wheat aphids per plant in the growing season of 2013–2014. Wheat was planted on dates (A) 24^th^ November (B) 27^th^ November, (C) 3^rd^ December. Control: no spray; MLE: spray of moringa leaf extract; MLE+NSE: spray of moringa leaf extract in combinaiton with neem seed extract; NSE-1: one spray of neem seed extract; I: spray of imidacloprid; I+MLE: spray of imidcloprid in combination with moringa leaf extract; NSE-2: two sprays of neem seed extract. Means on a given sowing date sharing common letters are not statistically different at *P* < 0.05 (Tukey’s HSD test).

Seasonal mean (±SEM) numbers of wheat aphid per plant in wheat field with seven treatments are presented in Figs 2 and 3. Mean number of wheat aphids per plant responded significantly to various treatments in November 27^th^ (*F*_6,14_ = 32.93, *P*<0.001), December 6^th^ (*F*_6,14_ = 166.27, *P*<0.001) and December 16^th^ (*F*_6,14_ = 423.32, *P*<0.001) in planted wheat crops during the season 2012–13; and November 24^th^ (*F*_6,14_ = 37.92, *P*<0.001), November 27^th^ (*F*_6,14_ = 56.75, *P*<0.001) and December 3^rd^ (*F*_6,14_ = 14.51, *P*<0.001) during season 2013–14. In 2012–13, aphid infestation was similar between MLE and control plots in November 27^th^ and December 6^th^ wheat while comparatively less numbers of aphids were recorded in MLE compared to the control in December 16^th^ planted wheat (Fig 2). NSE-1/2 significantly reduced pest numbers compared to MLE and the control; however, imidacloprid with or without MLE was the most effective in suppressing pest infestations (Figs 2 and 3). The effects of two applications of NSE on wheat aphid suppression were statistically at par compared to NSE one sprays with or without MLE in 2012–13 (Fig 2B and 2C). In 2013–14 experiments, imidacloprid with or without MLE was generally the most effective in lowering pest infestation followed by NSE. However, unlike 2012–13 experiments, aphid numbers in NSE two sprays plots were not significantly different from NSE one spray either alone or integrated with MLE in 2013–14 (Fig 3). Moreover, plots treated with MLE were less infested than the control in the wheat crops sowed during the earlier seasons (Fig 3A and 3B).

Inconsistency in the efficacy of botanical insecticides was observed between different sowing times. In November 27^th^ planted wheat, the aphid population densities showed no difference among the control, MLE, NSE-1 and MLE+NSE treatments, which may result from the asynchrony between peak aphid activity and the timing of insecticide applications. Aphid reached highest activity one week after the insecticide application (Figure A in S1 File). In December 6^th^ planted wheat, NSE and NSE+MLE exerted greater control of aphids compared to MLE and the control. This may be due to higher synchronization between the peak of aphid activity and insecticide application timing compared to the earlier case (Figure B in S1 File). In December 16^th^ planted wheat, NSE significantly suppressed aphid population as compared to MLE (well synchronized) (Figure C in S1 File). However, MLE remained more consistent in suppressing aphid population density than the control possibly due to short sowing times intervals and less aphid exposure in 2013–14 (Fig 3A and 3B).

### Natural enemy densities and predator-prey ratios

Table 3 presents population densities of natural enemies and predator–prey ratios during 2013–14 wheat growing season. In general, coccinellids were more abundant in untreated control in the wheat planted on November 24^th^ (*F*_6,14_ = 3.02, *P* = 0.041) and November 27^th^ (*F*_6,14_ = 16.68, *P*<0.001); however, they were more abundant under the treatment NSE-2 in the wheat planted on December 3^rd^ (*F*_6,14_ = 4.17, *P* = 0.013). Syrphids and spiders densities were more abundant in NSE-2 treated plots compared to other treatments in November 24^th^ (syrphids: *F*_6,14_ = 2.57, *P* = 0.006; spiders: *F*_6,14_ = 1.10, *P* = 0.40), November 27^th^ (syrphids: *F*_6,14_ = 4.93, *P* = 0.007; spiders: *F*_6,14_ = 3.00, *P* = 0.042) and December 3^rd^ (syrphids: *F*_6,14_ = 7.56, *P* = 0.001; spiders: *F*_6,14_ = 2.61, *P* = 0.06). Numerically, aphid mummies per plant were more abundant under the treatment NSE-2 than other treatments in November 24^th^ (*F*_6,14_ = 6.11, *P* = 0.003) and November 27^th^ (*F*_6,14_ = 1.31, *P* = 0.31). Moreover, ratios of spiders and parasitoids to their prey aphids did not differ among treatments in 2013. Predator prey ratios had significant difference among treatments only in November 27^th^ (coccinellids: *F*_6,14_ = 3.54, *P* = 0.024) and December 3^rd^ (syrphids: *F*_6,14_ = 4.74, *P* = 0.008) planted wheat.

**Table 3:** Mean (± SEM) seasonal sum of predators and predator–prey ratios per plot from wheat fields planted at three different dates during the growing season of 2013–14.

**24-November**								
		**Control**	**MLE**	**MLE+NSE**	**NSE-1**	**I**	**I+MLE**	**NSE-2**
Coccinellids	Number^b^	6.31 ± 1.39 a	2.93 ± 0.55 ab	3.55 ± 0.32 ab	5.33 ± 2.03 ab	0.62 ± 0.38 b	1.86 ± 0.55 ab	4.44 ± 1.43 ab
	Ratio^c^	0.085 ± 0.020 a	0.055 ± 0.010 a	0.102 ± 0.004 a	0.120 ± 0.040 a	0.060 ± 0.035 a	0.137 ± 0.085 a	0.142 ± 0.053 a
Spiders	Number	0.31 ± 0.11 a	0.13 ± 0.07 a	0.35 ± 0.08 a	0.35 ± 0.170 a	0.35 ± 0.17 a	0.26 ± 0.15 a	1.06 ± 0.67 a
	Ratio	0.004 ± 0.001 a	0.002 ± 0.001 a	0.010 ± 0.002 a	0.008 ± 0.004 a	0.032 ± 0.016 a	0.016 ± 0.008 a	0.035 ± 0.022 a
Syrphids	Number	0.53 ± 0.27 a	0.13 ± 0.07 a	0.88 ± 0.35 a	0.71 ± 0.32 a	0.44 ± 0.08 a	0.35 ± 0.23 a	1.86 ± 0.70 a
	Ratio	0.007 ± 0.003 a	0.003 ± 0.001 a	0.025 ± 0.009 a	0.016 ± 0.006 a	0.040 ± 0.006 a	0.020 ± 0.010 a	0.054 ± 0.023 a
Parasitoids^d^	Number	4.62 ± 0.62 a	1.33 ± 0.67 b	3.46 ± 0.70 ab	1.42 ± 0.84 b	1.06 ± 0.53 b	1.06 ± 0.40 b	3.73 ± 0.26 ab
	Ratio	0.062 ± 0.010 a	0.027 ± 0.013 a	0.099 ± 0.017 a	0.032 ± 0.018 a	0.091 ± 0.046 a	0.071 ± 0.037 a	0.109 ± 0.006 a
**27-November**								
Coccinellids	Number	4.13 ± 0.20 a	0.22 ± 0.16 b	1.82 ± 0.42 b	4.08 ± 0.75 a	0.8 ± 0.30 b	0.97 ± 0.17 b	4.00 ± 0.55 a
	Ratio	0.045 ± 0.005 ab	0.004 ± 0.002 b	0.058 ± 0.010 ab	0.113 ± 0.040 a	0.067 ± 0.023 ab	0.089 ± 0.024 ab	0.128 ± 0.019 a
Spiders	Number	0.13 ± 0.07 b	0.13 ± 0.07 b	0.44 ± 0.08 ab	0.31 ± 0.16 ab	0.53 ± 0.15 ab	0.44 ± 0.17 ab	1.42 ± 0.64 a
	Ratio	0.002 ± 0.0009 a	0.002 ± 0.001 a	0.014 ± 0.003 a	0.009 ± 0.005 a	0.045 ± 0.012 a	0.038 ± 0.013 a	0.047 ± 0.021 a
Syrphids	Number	0.22 ± 0.08 b	0.08 ± 0.08 b	0.22 ± 0.04 b	0.48 ± 0.11 b	0.26 ± 0.15 b	0.44 ± 0.17 b	2.60 ± 1.07 a
	Ratio	0.003 ± 0.001 a	0.002 ± 0.001 a	0.007 ± 0.001 a	0.013 ± 0.004 a	0.023 ± 0.013 a	0.042 ± 0.020 a	0.084 ± 0.036 a
Parasitoids	Number	1.42 ± 0.77 a	1.60 ± 0.85 a	1.95 ± 1.45 a	3.02 ± 1.24 a	1.77 ± 0.58 a	0.97 ± 0.84 a	4.44 ± 1.17 a
	Ratio	0.017 ± 0.008 a	0.028 ± 0.014 a	0.055 ± 0.040 a	0.087 ± 0.048 a	0.156 ± 0.058 a	0.080 ± 0.069 a	0.143 ± 0.039 a
**3-December**								
Coccinellids	Number	2.93 ± 0.55 a	2.17 ± 0.65 ab	2.31 ± 0.17 ab	2.48 ± 0.23 ab	1.24 ± 0.08 b	1.24 ± 0.32 b	3.17 ± 0.13 a
	Ratio	0.042 ± 0.007 a	0.044 ± 0.010 a	0.068 ± 0.007 a	0.066 ± 0.006 a	0.130 ± 0.012 a	0.218 ± 0.124 a	0.128 ± 0.006 a
Spiders	Number	0.17 ± 0.08 a	0.08 ± 0.04 a	0.71 ± 0.23 a	0.53 ± 0.15 a	0.35 ± 0.08 a	0.44 ± 0.23 a	1.77 ± 0.84 a
	Ratio	0.003 ± 0.001 a	0.002 ± 0.001 a	0.022 ± 0.007 a	0.013 ± 0.002 a	0.038 ± 0.011 a	0.045 ± 0.023 a	0.073 ± 0.035 a
Syrphids	Number	0.88 ± 0.51 b	0.17 ± 0.11 b	0.71 ± 0.35 b	0.95 ± 0.18 b	0.33 ± 0.24 b	0.42 ± 0.08 b	2.82 ± 0.48 a
	Ratio	0.015 ± 0.010 b	0.004 ± 0.002 b	0.020 ± 0.010 b	0.028 ± 0.009 b	0.037 ± 0.027 ab	0.066 ± 0.026 ab	0.110 ± 0.017 a
Parasitoids	Number	4.62 ± 1.66 a	1.28 ± 0.77 a	5.06 ± 0.96 a	5.06 ± 1.38 a	1.68 ± 0.17 a	1.24 ± 0.64 a	3.11 ± 0.77 a
	Ratio	0.072 ± 0.031 a	0.035 ± 0.021 a	0.147 ± 0.021 a	0.137 ± 0.037 a	0.177 ± 0.022 a	0.125 ± 0.064 a	0.125 ± 0.029 a

^a^ Means in rows sharing common letters are not different at *P* < 0.05 (Tukey’s HSD test).

^b^ Means per plant,

^c^ Predator-prey ratios,

^d^ Mean number of mummified aphids per plant.

### Comparative effect of imidacloprid and botanicals on wheat yield

Average yield in terms of shoot biomass, grain number per spike and thousand grain weight are presented in Tables 4 and 5. During 2012–13, shoot biomass did not differ significantly among the treatments within each harvest (November 27^th^: *P* = 0.085; December 6^th^: *P* = 0.066; December 16^th^: *P* = 0.056). Grain numbers per spike differed markedly among the treatments within each harvest (all *P*<0.001) (Table 4). Thousand grain weights responded significantly to the treatments during the harvests except for the ones planted on December 16^th^ (*P* = 0.11) (Table 4). Highest grain numbers per spike and thousand grain weights were produced from the plots treated with I+MLE whereas the lowest production was obtained from the control plots (Table 4). Percentage yield differences in terms of grain number per spike (41.51–81.89%) and thousand grain weights (6.50–13.88%) were obtained from the plots treated with I+MLE over the control. MLE plots had significantly more wheat grain numbers per spike (10.05–18.61%) and thousand grain weights (4.90–8.41%) over the control (Table 4). Treatments with NSE showed variable results. In at least two out of three trials in 2012–13, grains numbers per spike were more in plots receiving two applications rather than one application either alone or integrated with MLE. However, grain weight did not differ significantly between I and NSE either alone or in integration with MLE (Table 4).

**Table 4:** Effect of various treatments on shoot biomass, grains/spike and thousand grains weights per plot from wheat planted on different dates during 2012–13 wheat growing season.

Sowing date	Treatments	Shoot biomass (g)	Grains/spike	Grain weight (g)
		Mean	Mean	Mean
27-Nov	Control	3.78±0.20 a	28.83±0.26 g	43.85±0.22 d
	MLE	4.12±0.18 a	31.73±0.29 f	46.00±0.42 cd
	MLE+NSE	4.07±0.16 a	37.76±0.24 d	48.72±0.39 ab
	NSE-1	3.83±0.42 a	34.60±0.26 e	46.61±0.77 bc
	Imidacloprid(I)	4.07±0.23 a	44.76±0.53 b	46.47±0.07 c
	I+MLE	3.60±0.36 a	47.10±0.25 a	49.65±0.34 a
	NSE-2	3.96±0.30 a	41.20±0.36 c	49.94±0.21 a
6-Dec	Control	3.24±0.17 a	34.06±1.82 d	44.55±0.28 b
	MLE	3.55±0.13 a	40.40±0.30 c	48.30±0.61 a
	MLE+NSE	3.56±0.35 a	42.33±0.69 bc	49.01±0.54 a
	NSE-1	3.16±0.19 a	44.06±0.43 b	49.16±0.79 a
	Imidacloprid(I)	3.65±0.20 a	44.43±0.08 b	48.34±0.50 a
	I+MLE	3.56±0.19 a	48.20±0.26 a	49.35±0.55 a
	NSE-2	3.38±0.25 a	42.93±0.60 bc	47.85±0.70 a
16-Dec	Control	3.99±0.43 a	25.80±0.30 g	45.2±0.41 a
	MLE	3.88±0.42 a	28.76±0.27 f	47.8±0.65 a
	MLE+NSE	4.11±0.11 a	35.46±0.55 d	47.57±0.61 a
	NSE-1	3.54±0.31 a	32.00±0.51 e	47.76±1.29 a
	Imidacloprid(I)	3.62±0.25 a	42.33±0.70 b	47.71±0.63 a
	I+MLE	3.87±0.37 a	46.93±0.40 a	47.61±0.22 a
	NSE-2	4.40±0.17 a	38.43±0.33 c	48.14±0.32 a

Means in column within a sowing date sharing common letters are not different at *P* < 0.05 (Tukey’s HSD test).

**Table 5:** Effect of various treatments on shoot biomass, grains/spike and thousand grains weights per plot from wheat planted on different dates during 2013–14 wheat growing season.

Sowing date	Treatments	Shoot biomass (g)	Grains/spike	Grain weight (g)
		Mean	Mean	Mean
24- Nov	Control	3.03±0.06 c	30.66±0.66 d	41.86±0.34 e
	MLE	3.73±0.03 b	35.33±1.20 bcd	44.97±0.11 de
	MLE+NSE	5.70±0.11 a	38.33±1.20 abc	48.17±0.64 cd
	NSE-1	5.83±0.03 a	34.33±0.88 cd	46.08±1.43 d
	Imidacloprid(I)	5.63±0.14 a	39.66±0.88 ab	52.08±0.34 ab
	I+MLE	5.86±0.03 a	41.33±1.45 a	55.49±0.83 a
	NSE-2	5.76±0.06 a	35.00±1.52 bcd	49.55±0.30 bc
27- Nov	Control	2.80±0.05 c	31.33±1.20 a	39.35±0.28 f
	MLE	3.46±0.08 b	31.33±0.88 a	42.83±0.17 e
	MLE+NSE	5.80±0.05 a	34.66±2.33 a	46.40±0.58 cd
	NSE-1	5.90±0.05 a	32.66±0.66 a	45.33±0.61 de
	Imidacloprid(I)	5.63±0.08 a	35.00±2.08 a	50.09±0.22 b
	I+MLE	5.56±0.12 a	37.33±2.02 a	53.25±1.03 a
	NSE-2	5.60±0.20 a	33.00±0.57 a	48.52±0.24 bc
3- Dec	Control	2.60±0.17 c	30.33±0.66 bc	38.30±0.66 e
	MLE	3.16±0.06 b	32.00±0.57 bc	41.14±0.32 d
	MLE+NSE	5.53±0.03 a	32.66±0.33 bc	45.24±0.45 c
	NSE-1	5.96±0.06 a	31.66±0.88 bc	44.76±0.67 c
	Imidacloprid(I)	5.50±0.05 a	35.00±1.00 ab	49.80±0.32 b
	I+MLE	5.86±0.03 a	38.66±0.88 a	52.71±0.77 a
	NSE-2	5.50±0.15 a	29.33±1.76 c	46.97±0.76 c

Means in column within a sowing date sharing common letters are not different at *P* < 0.05 (Tukey’s HSD test).

Yield kg/ha was significantly different among plots with various treatments for the wheat planted on November 27^th^ (*P*<0.001), December 6^th^ (*P*<0.001) and December 16^th^ (*P*<0.001). Percentage differences in yield kg/ha over the control for treatments were as follows: I+MLE (27.59–39.03%), MLE+NSE (7.91–51.75%), NSE-2 (19.50–52.74%) and NSE-1 (9.89–40.50%) in 2012–13.

During 2013–14, ANOVA revealed significant differences for shoot biomass (*P*<0.001), grain numbers per spike (*P*<0.001) and thousand grain weights (*P*<0.001) among treatments in each experiment. Shoot biomass, grains number per spike and thousand grain weights were higher in I+MLE and lowest in the control plots (Table 5). Application of MLE significantly increased thousand grain weights compared to the control. Percentage yield difference in terms of thousand grain weight for MLE over the control ranged between 7.41–8.84%. The similar thousand grain weights were obtained from the MLE+NSE treatment and NSE-2 treatment in all the experiments during 2013–14 (Table 5). Percentage yield differences for MLE+NSE and NSE-2 in terms of thousand grain weight over the control were recorded as 15.07–18.12% and 18.37–23.30%, respectively.

Yield kg/ha was significant among plots treated with I and NSE with or without MLE in November 24^th^ (*P*< 0.001), November 27^th^ (*P*< 0.001) and December 3^rd^ (*P*< 0.001). Percentage differences in yield kg/ha after addition of MLE in imidacloprid and NSE, compared to the control were as follows: MLE plots (10.43–18.00%); MLE+NSE (36.58–38.92%) and I+MLE (53.20–61.12%).

## Discussion

Wheat aphids, *S*. *graminum*, *R*. *padi* and *S*. *avenae* have become routine pests of wheat since last two decades and pose major threat to wheat production in Pakistan. Integrated management strategies against aphids such as insecticides are of utmost importance to reduce economic losses in wheat [36]. Synthetic insecticides have been found effective in managing wheat aphid and thus minimizing yield losses. However, the accumulation of toxic compounds in the grain makes it a less desirable constituent for aphid management, particularly for wheat, i.e. a staple food in Pakistan. Our data clearly demonstrated that imidacloprid was the most effective in suppressing aphid population, followed by the NSE (Figs 2 and 3). Overall, the order of efficiency in reducing aphid populations was imidacloprid (I) >NSE in the three out of six experiments, while I >MLE in the six out of six experiments. Aphids were more effectively controlled with imidacloprid spraying compared to botanicals (one or two sprays) with one application on heading stage till end March.

We recorded non-significant difference in aphid population density between the treatment imidacloprid (alone or with MLE) and the treatment NSE (alone, with MLE, or two sprays) in at least three out of six experiments. Variability in effectiveness of NSE in experiments may be attributed to the changes in environmental conditions and crop variety [48]. Under laboratory conditions, neem seed oil and neem seed extracts were as effective as botanical pyrethrum were able to suppress green peach aphid, lettuce aphid as well as strawberry aphids on pepper and strawberry, but not lettuce [48]. Toxicity and effectiveness of NSE can be differential with respect to activity of other active ingredients in addition to pure azadirachtin for example; a value of 0.674% LC_50_ for neem seed kernel extracted in hexane was reduced to 0.328% LC_50_ when the former extract was partitioned with the neem seed extracted in ethanol against the mustard aphid, *Lipaphis erysimi* (Kalt.) (Hemiptera: Aphididae). Ethanol soluble fraction of this partitioned extract had eight different compounds including azadirachtin [73]. Moreover, the type of formulation, extraction methods and environmental conditions could be responsible for differential effects of neem products [37]. Various studies have documented the effective use of neem formulations for the control of aphids [74–76]. Neem based formulation not only shows systemic and contact actions but also acts as antifeedent, sterilents, growth inhibitors and toxicological repellents against insect pests, which has been considered as a low-cost management tactic [77].

In our study, the aphid density per plant was lower in MLE plots than the control in three out of six experiments. The low densities of aphids in MLE plots may be attributed to insecticidal potential of *M*. *oleifera* that contain lectins like WSMoL (water-soluble *M*. *oleifera* lectin) [78]. *M*. *oleifera* lectins could bind with the chitin and reported to have larvicidal effect on *Aedes aegypti* L. (Diptera: Culicidae) [78]. Literature is scarce about the insecticidal activity of MLE; however, in field studies, MLE reduced aphid infestation compared to the control when applied solely. Moreover, the populations of aphid were more effectively suppressed when it was applied in combination with neem and eucalyptus leaves extract, *Eucalyptus globulus* Labill (family Myrtaceae) [79, 80].

In our findings, inconsistency in the efficacy of botanical insecticides was mainly due to aphid preference among sowing dates which created asynchrony between the peak of aphid activity and insecticide application timing. The synchronization between the peak aphid activity and insecticide application timings is the key for effective pest control [81]. Further, insecticide efficacy to pest insects may vary to wheat variety resistance and susceptibility traits [82], and environmental conditions (S1 and S2 Files) [83]. Our findings regarding aphid preference among sowing dates were in consistence with Chander et al. [84] who reported that aphids preferred the late-sown wheat at vegetative growth stage than the timely-planted wheat at the reproductive stage.

In Pakistan, management of wheat aphids relied heavily on natural enemies. Biocontrol agents such as parasitoids, lady beetles, hover flies, green lacewing and spiders can considerably contribute to the pest management worldwide [71, 85–90]. However, predator efficiency and their development is affected by various factors such as competency for prey resource, intraguild predation [91] and/or temperature [92]. In our findings, population densities of coccinellids and parasitoids were the lowest in imidacloprid-treated plots and the ratio of coccinellids, parasitoids and spiders to their prey aphid were not influenced by imidacloprid. Lower abundance of coccinellids and parasitoids may result from the decreased availability of aphid prey, and the predators are assumed to emigrate to search for other food sources. Food resource is an important factor for females to decide whether oviposit or not at the site [93]. In addition, the negative effect of neonicotinoids insecticides such as imidacloprid and thiamethoxam on four parasitoid and two predator species in a systemic uptake bioassay has been reported by Prabhaker et al. [94]. Imidacloprid at the sublethal concentration of LC5 and 10% LC5 shortened adult *Coccinella septempunctata* L. (Coleoptera: Coccinellidae) longevity by 23.97 and 28.68%, and reduced the fecundity by 52.81 and 56.09% in comparison to the control populations [95]. Neonicotinoids have negative impact on non-target animals in natural and agricultural ecosystem [96]. For example, Douglas et al. [97] reported that soybean seeds treated with thiamethoxam, a neonicotinoid, had no effect on pest slug, *Deroceras reticulatum* Muller (Pulmonata: Agriolimacidae), but the toxin was transmitted to the predacious beetle, *Chlaenius tricolor* Dejean (Coleoptera: Carabidae) resulting in >60% mortality or impairment. Field collected slug contained up to 500 ng g^-1^ of neonicotinoid residues which could harm their predator insects [97]. Chronic exposure to imidacloprid at higher field doses ranging between 20 and 100μg/kg in pollen could had negative impact on health as well overwintering success of bees colony [98] and affect immunocompetence response of bees towards diseases [99]. Imidacloprid concentrations (20 ng/L) in surface water negatively correlated with insectivorous bird’s abundance and reduced their annual average population to 3.5% [100]. However, application of imidacloprid is recommended with caution as several agricultural pests worldwide have been reported to develop resistance [101, 102]. Moreover, nicotinic insecticide like imidacloprid should be rotated with botanicals to delay the development of resistance, as the rotation of chemicals with unrelated mode of action is the key stone strategy to manage resistance development in insects [103]. Self-prepared formulations of neem like NSE could be more advantageous over their commercial formulation, owing to the presence of multiple active ingredients in the former compared to latter that contain pure active ingredient such as azadirachtin. Blend of active substance in NSE may exhibit various mode of action and can inhibit detoxification mechanism specific for azadirachtin, which may diffuse the selection process and mitigate the development of resistance compared to single active ingredient formulation [104].

The current study showed that the mean densities of coccinellids, spiders, syrphids and parasitoids per plant were more abundant in NSE plots especially in NSE-2 compared to imidacloprid treated plots. Avoiding the direct ingestion of neem formulations by predators could be the reason of insensitivity to these formulations. Neem formulations were reported to be relatively safer than imidacloprid for wheat aphid parasitoids (mummified aphids) under field conditions [37]. Spray of neem seed oil and neem seed extracts did not reduce the parasitism rate of *Diaeretiella rapae* (McIntosh) (Hymenoptera: Braconidae) on green peach aphid, *M*. *persicae* under laboratory and field conditions [105].

**Fig 4:**
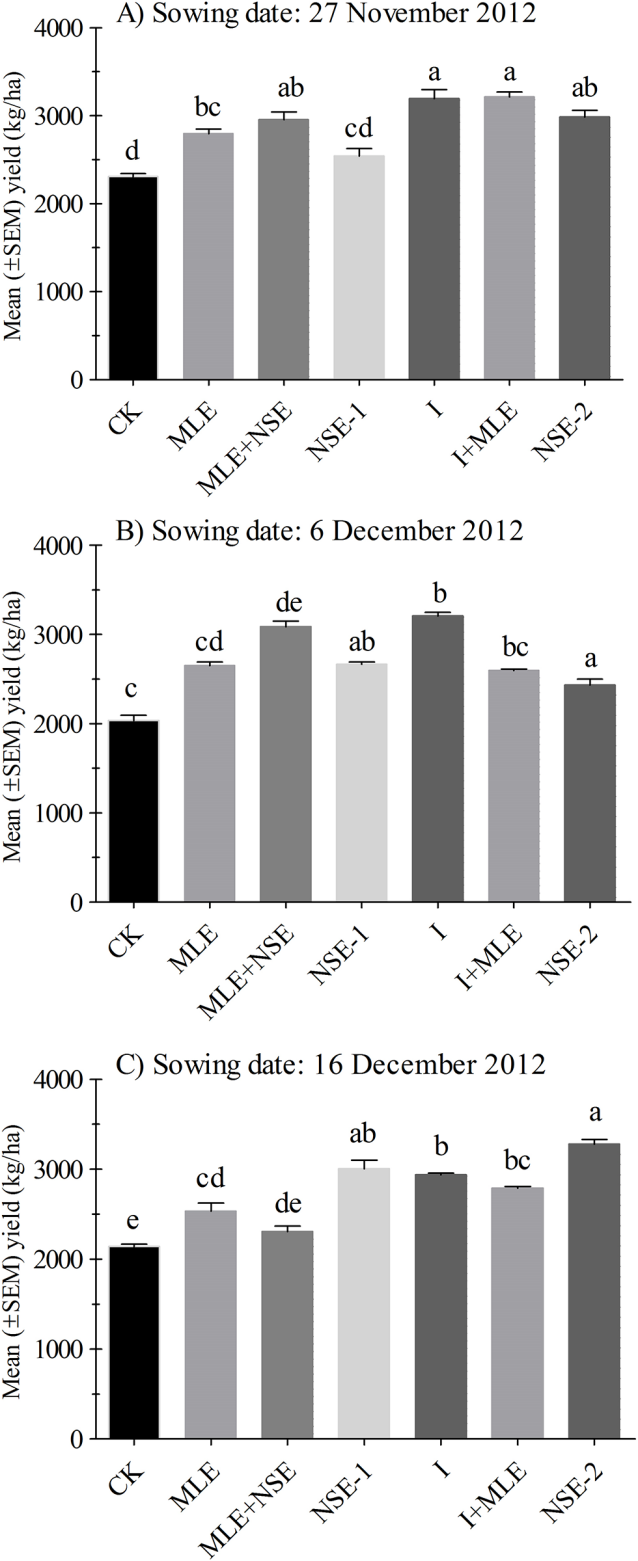
Mean (± SEM) yield (kg/ha) in 2012. Wheat was planted on dates (A) 27^th^ November (B) 6^th^ December, (C) 16^th^ December. Control: no spray; MLE: spray of moringa leaf extract; MLE+NSE: spray of moringa leaf extract in combinaiton with neem seed extract; NSE-1: one spray of neem seed extract; I: spray of imidacloprid; I+MLE: spray of imidcloprid in combination with moringa leaf extract; NSE-2: two sprays of neem seed extract. Means on a given sowing date sharing common letters are not statistically different at *P* < 0.05 (Tukey’s HSD test).

**Fig 5:**
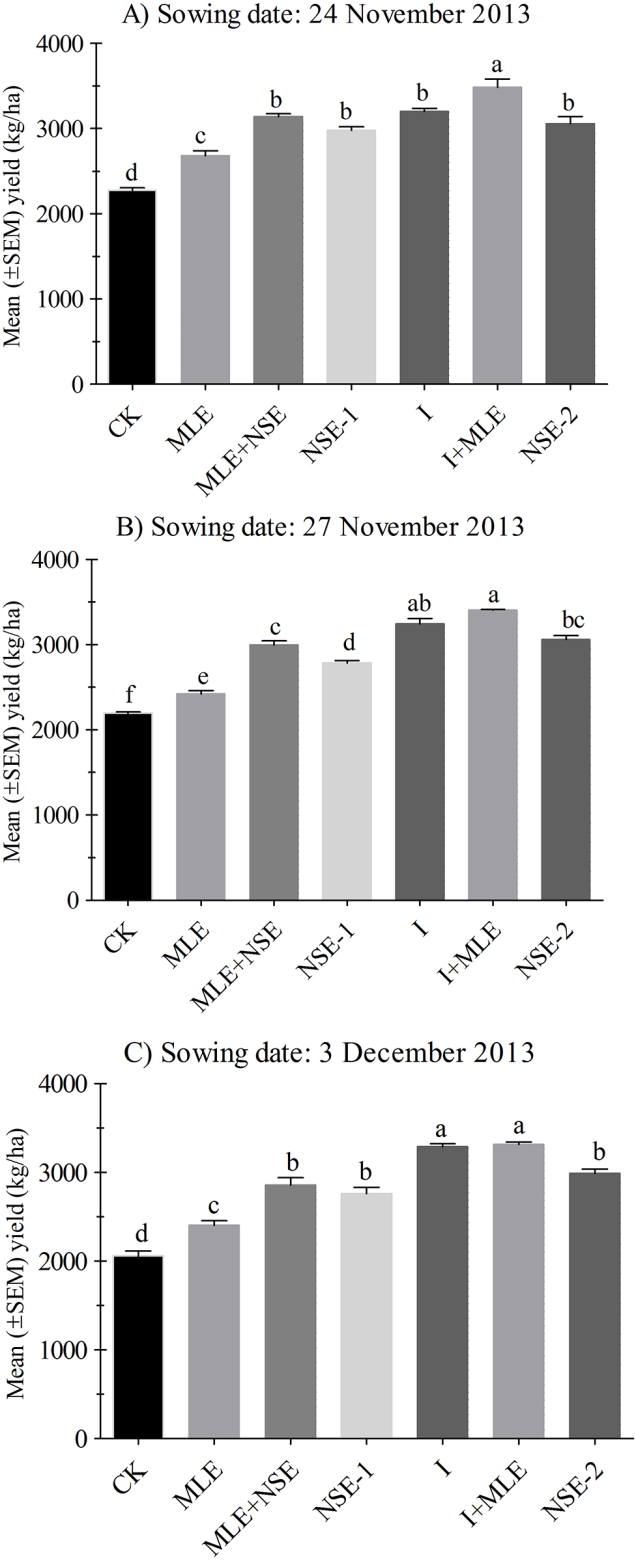
Mean (± SEM) yield (kg/ha) in 2013. Wheat was planted on dates (A) 24^th^ November (B) 27^th^ November, (C) 3^rd^ December. Control: no spray; MLE: spray of moringa leaf extract; MLE+NSE: spray of moringa leaf extract in combinaiton with neem seed extract; NSE-1: one spray of neem seed extract; I: spray of imidacloprid; I+MLE: spray of imidcloprid in combination with moringa leaf extract; NSE-2: two sprays of neem seed extract. Means on a given sowing date sharing common letters are not statistically different at *P* < 0.05 (Tukey’s HSD test).

Yield and related parameters were significantly improved by single foliar application of MLE at heading compared to control. Moreover, addition of MLE into both imidacloprid and NSE treatment greatly improved wheat yield in almost all the trials. The positive effect of MLE application on the yield by suppressing aphid population may be due to the presence of growth promoting substances “Zeatin”, a natural cytokinins, that delays early maturity [106], as well as the presence of ascorbates, phenols carotenoids, potassium and calcium in MLE that are known to stimulate growth [107]. Consequently, yield parameters of late planted wheat could be enhanced [61]. In another report, seed priming with MLE diluted 30 times with distilled water improved seedling emergence and growth in maize due to more chlorophyll and phenolics contents in seedling [68]. Moreover, when NSE and imidacloprid were integrated with MLE, their yield potential was enhanced than MLE alone in most of trials. This may be due to low pest pressure and additionally the MLE increased grain filling periods which produced more yield than MLE sole. Moreover, wheat yield under the treatments NSE (alone, with MLE, or two sprays) were comparable to those under imidacloprid (with or without MLE). This further confirms that in late planted wheat, it is the aphid, other than early rise in temperature that causes losses to wheat yield. Therefore, the plots with minimum aphid infestation were able to produce more yield kg/ha (Figs 4 and 5).

In summary, our data demonstrated a strong efficiency of imidacloprid in wheat aphid management. The second most efficient tactic for the wheat aphid management was NSE. However, natural enemies including coccinellids, spiders, syrphids and parasitoids were more abundant in NSE plots compared to imidacloprid-treated ones. Moreover, yields losses in the plots protected by NSE treatment were considerably comparable to those treated by imidacloprid. While considering the efficacy of NSE along with MLE, yield losses are limited due to aphids and temperature stress in late sown wheat. Therefore, we recommend the combined use of NSE and MLE particularly in late sown wheat. However, moderate effect of NSE against aphids can compel farmers to give multiple applications in which biopesticides may induce sublethal impacts on natural enemies [15]. Further research on degradation dynamics of the bio insecticides in wheat fields and their sub-lethal impact on natural enemies would be helpful in integrating these promising plant based insecticides with biological control agents for aphid management.

## Supporting information

S1 File Mean (± SE) number of wheat aphids per plant on different observation dates in the growing season 2012–2013. Wheat was planted on dates (A) 27^th^ November (B) 6^th^ December, (C) 16^th^ December. Control: no spray; MLE: spray of moringa leaf extract; MLE+NSE: spray of moringa leaf extract in combinaiton with neem seed extract; NSE-1: one spray of neem seed extract; I: spray of imidacloprid; I+MLE: spray of imidcloprid in combination with moringa leaf extract; NSE-2: two sprays of neem seed extract. Means on a given sowing date sharing common alphabets are not statistically different at *P* < 0.05 (Tukey’s HSD test).
(PDF)

S2 File Mean (± SE) number of wheat aphids per plant on different observation dates in the growing season 2013–14. Wheat was planted on dates (A) 24^th^ November (B) 27^th^ November, (C) 3^rd^ December. Control: no spray; MLE: spray of moringa leaf extract; MLE+NSE: spray of moringa leaf extract in combinaiton with neem seed extract; NSE-1: one spray of neem seed extract; I: spray of imidacloprid; I+MLE: spray of imidcloprid in combination with moringa leaf extract; NSE-2: two sprays of neem seed extract. Means on a given sowing date sharing common alphabets are not statistically different at *P* < 0.05 (Tukey’s HSD test).
(PDF)
