# Supplementary figures and images for: Comparative role of neem seed extract, moringa leaf extract and imidacloprid in the management of wheat aphids in relation to yield losses in Pakistan
Source: PLoS One. 2017 Sep 27;12(9):e0184639. doi: 10.1371/journal.pone.0184639 (PMC5617159; doi:10.1371/journal.pone.0184639)

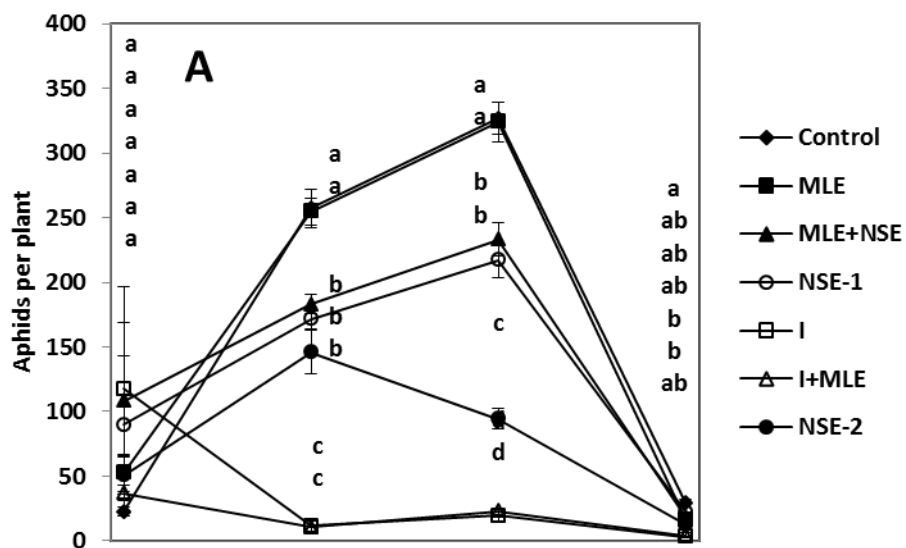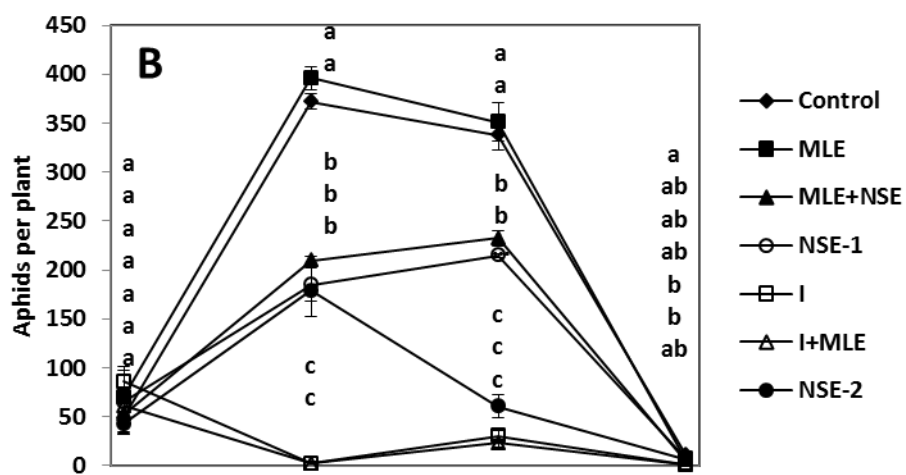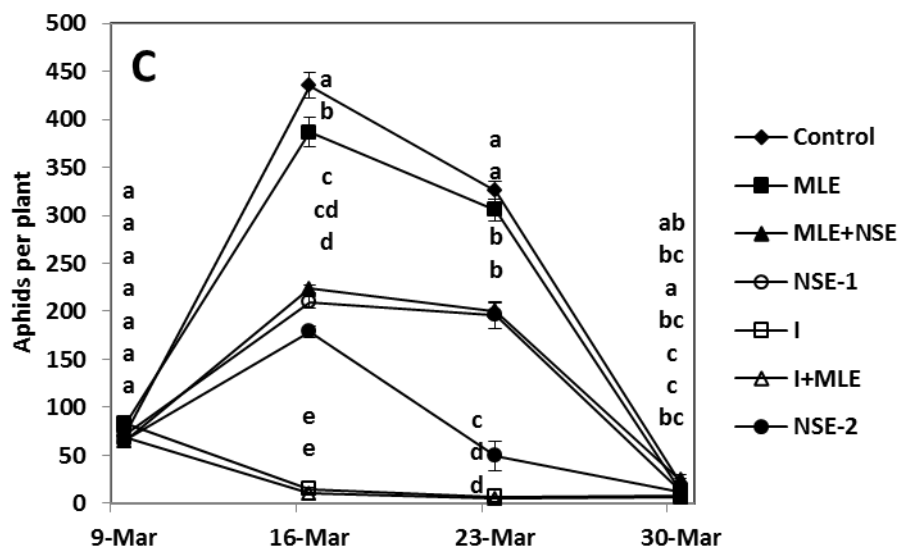

Supplement: S1 File — Wheat was planted on dates (A) 27th November (B) 6th December, (C) 16th December. Control: no spray; MLE: spray of moringa leaf extract; MLE+NSE: spray of moringa leaf extract in combinaiton with neem seed extract; NSE-1: one spray of neem seed extract; I: spray of imidacloprid; I+MLE: spray of imidcloprid in combination with moringa leaf extract; NSE-2: two sprays of neem seed extract. Means on a given sowing date sharing common alphabets are not statistically different at P < 0.05 (Tukey’s HSD test). (PDF) [file pone.0184639.s001.pdf]

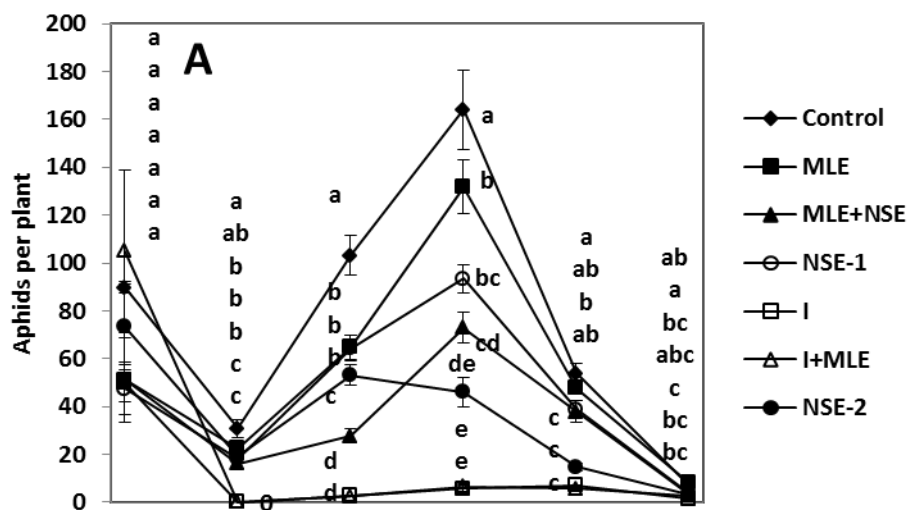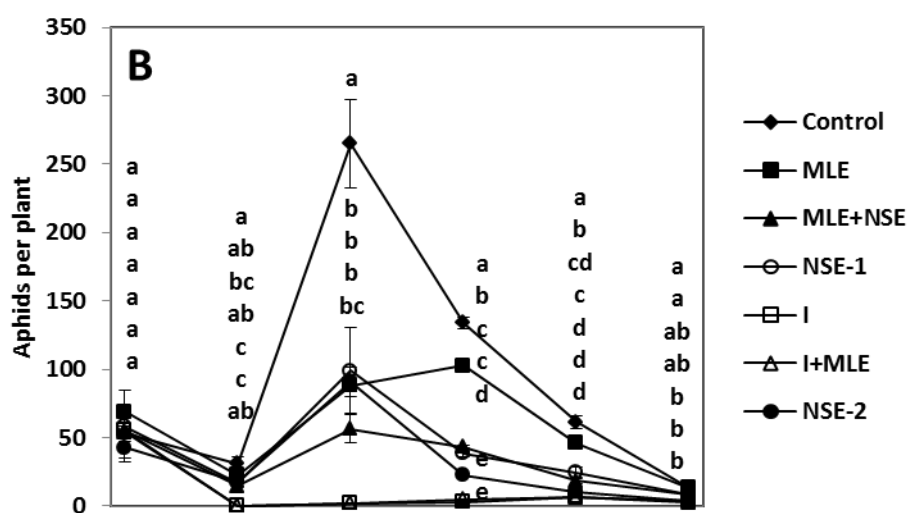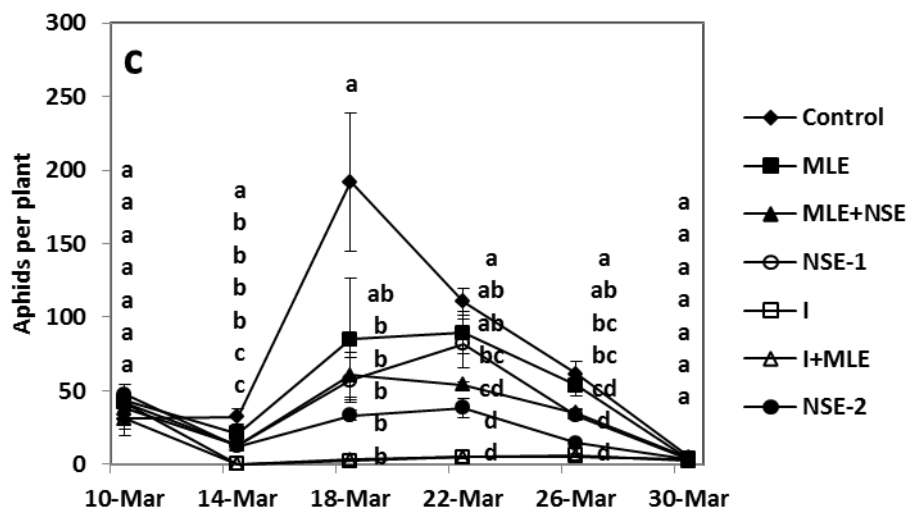

Supplement: S2 File — Wheat was planted on dates (A) 24th November (B) 27th November, (C) 3rd December. Control: no spray; MLE: spray of moringa leaf extract; MLE+NSE: spray of moringa leaf extract in combinaiton with neem seed extract; NSE-1: one spray of neem seed extract; I: spray of imidacloprid; I+MLE: spray of imidcloprid in combination with moringa leaf extract; NSE-2: two sprays of neem seed extract. Means on a given sowing date sharing common alphabets are not statistically different at P < 0.05 (Tukey’s HSD test). (PDF) [file pone.0184639.s002.pdf]
